# Hedgehog−Gli2 Signaling Promotes Chemoresistance in Ovarian Cancer Cells by Regulating MDR1

**DOI:** 10.3389/fonc.2021.794959

**Published:** 2022-01-04

**Authors:** Qian Wang, Xin Wei, Lanyan Hu, Lingling Zhuang, Hong Zhang, Qi Chen

**Affiliations:** Department of Gynecology and Obstetrics, The Second Affiliated Hospital of Nanchang University, Nanchang, China

**Keywords:** cisplatin resistance, Gli2, hedgehog signaling, MDR1, ovarian cancer, proliferation

## Abstract

**Background:**

Cisplatin (DDP) resistance remains a key challenge in improving the clinical outcome of patients with ovarian cancer (OC). *Gli2* overexpression can lead to DDP resistance in OC cells, but the specific underlying regulatory mechanism remains unclear. The membrane transporter encoding gene *MDR1* positively regulates chemotherapy resistance in various cancer types. We evaluated *MDR1* as a potential *Gli2* downstream target and the contribution of the *Gli2/MDR1* axis in promoting DDP resistance in OC cells.

**Methods:**

To generate drug-resistant SKOV3/DDP cells, SKOV3 cells were grown for six months under continuous induction wherein the DDP concentration was steadily increased. *Gli2* expression in OC cells with varying DDP sensitivities was detected using western blot. Cell counting kit-8 assays were used to assess the DDP sensitivity of SKOV3, SKOV3/DDP, A2780, and A2780/DDP cells and reversal of DDP resistance in SKOV3/DDP and A2780/DDP cells. Cell proliferation was analyzed using 5-ethynyl-2′-deoxyuridine (EdU) incorporation assays. The transcriptional regulation of *MDR1* by *Gli2* was determined using luciferase reporter assays. Finally, xenograft OC tumors were generated in nude mice, which were then treated with intraperitoneal DDP or phosphate-buffered saline (PBS) injections to investigate if *Gli2* affected DDP resistance in OC *in vivo*.

**Results:**

DDP-resistant SKOV3/DDP and A2780/DDP cells showed higher expression of *Gli2* and *MDR1* as compared with that in DDP-sensitive OC cells. *Gli2* knockdown in SKOV3/DDP cells significantly reduced *MDR1* expression, whereas it increased DNA damage, thereby sensitizing OC cells to DDP. Similar results were obtained after targeting Gli2 expression with the Gli-antagonist 61 inhibitor (GANT61) in SKOV3/DDP and A2780/DDP cells. In cells stably overexpressing *Gli2*, treatment with gradient concentrations of verapamil, an MDR1 inhibitor, significantly inhibited *MDR1* expression. Our findings indicate that downregulation of *MDR1* expression may reverse OC cell resistance to DDP. Moreover, dual-luciferase reporter gene assays confirmed that *MDR1* is a direct downstream target of *Gli2*, with *Gli2* positively regulating *MDR1* expression. Finally, subcutaneous xenotransplantation in nude mice demonstrated that *Gli2* plays a key role in regulating OC drug resistance.

**Conclusions:**

We identified a mechanism by which Hedgehog-*Gli* signaling regulates OC chemoresistance by modulating *MDR1* expression. Hence, *Gli2* and *MDR1* are potential biomarkers and therapeutic targets in patients with chemoresistant OC.

## Introduction

Ovarian cancer (OC) is the deadliest gynecological malignancy. For decades, cytoreductive surgery and platinum- or taxane-based combination chemotherapy have been the main treatment modalities for OC (1). Although the first response rate to first-line chemotherapy can be as high as 65–80%, drug resistance during later stages of chemotherapy leads to a high risk of recurrence, metastasis, and death (2–4). Chemoresistance is the most important clinical barrier in the treatment of OC (5). Particularly, cisplatin (DDP) resistance greatly impacts OC prognosis. Currently, platinum resistance is the primary challenge in treating OC (6, 7). Therefore, new biomarkers for early prediction of chemotherapy resistance and treatment strategies for reversing resistance are urgently needed.

In metazoans, the Hedgehog (Hh) signaling pathway is a critical regulator of embryonic development and homeostasis (8, 9). The Hh family in vertebrates consists of four main components: three secreted ligands (Sonic Hedgehog, Desert Hedgehog, and Indian Hedgehog), membrane receptor Patched (*PTCH1–2*), G-protein coupled transmembrane receptor Smoothened, and three glioma-associated oncogene (*Gli1–3*) transcription factors (10). In mammals, *Gli2* is required for embryonic development (11).

Previous reports showed that the absence of *Gli2* in mice can lead to embryonic death, whereas *Gli1* is dispensable for the development or survival of the animals (11). Moreover, Hh signals play a critical role in chemotherapy resistance in several tumor types, including gynecologic malignancies (12–15).

Chemotherapy efficacy is frequently limited by the development of multidrug resistance (MDR) in cancer cells (16, 17). Thus, MDR is a major challenge in achieving favorable cancer treatment outcomes. Classical MDR is linked to increased expression of ATP-binding cassette transporters, which play a role in drug detoxification and protect tissues from exogenous substances (18, 19). Expression of multidrug resistance protein 1 encoding gene (*MDR1*, also known as *ABCB1)*, which encodes MDR1 (also called P-glycoprotein), is one of the most important predictors of MDR to chemotherapy in several malignancies (20). During chemotherapy, tumors can acquire MDR against chemotherapy drugs by inducing the expression of *MDR1* (21–24), which in turn confers drug resistance and inhibits targeted chemotherapy-induced cytotoxicity. Therefore, *MDR1* activity can confer MDR in cancer cells and prevent the drug from reaching therapeutic concentrations in the target cells or organs, limiting its therapeutic effects.

Our previous studies revealed that compared with the parental strain, the Hh signaling pathway is abnormally activated, transcription factor *Gli2* is abnormally expressed, and resistance to DDP is enhanced in DDP-resistant OC cells. However, the detailed molecular mechanism underlying DDP resistance remained unclear. In this study, we explored the specific mechanism underlying *Gli2-*mediated DDP resistance in OC. This study provides a foundation for developing strategies to improve the effectiveness of DDP chemotherapy for OC.

## Materials and Methods

### Reagents and Antibodies

DDP was purchased from Calbiochem (San Diego, CA, USA). The MDR1 inhibitor verapamil was purchased from Merck KGaA (Darmstadt, Germany). Gli antagonist 61 (GANT61), dimethyl sulfoxide, and polyethylenimine transfection reagent (cat. no. HY 13901, P8340 and 408727) were purchased from Sigma-Aldrich (St. Louis, MO, USA). Lipofectamine 2000 transfection reagent, penicillin/streptomycin, RIPA buffer, and the bicinchoninic acid protein assay kit (cat. no. 11668019, 15140122, 89900 and 23225) were procured from Thermo Fisher Scientific (Waltham, MA, USA). The Cell Counting Kit-8 (CCK-8; cat. no. C6005) was purchased from Everbright USA, Inc (Redmond, WA, USA). Puromycin (cat. no. P823025) was purchased from Solarbio (Beijing, China), and doxycycline (DOX) was purchased from Sangon Biotech (Shanghai, China). The primary antibody against phosphohistone H2AX (Ser139) (cat. no. 2577) was supplied by Cell Signaling Technology (Danvers, MA, USA). Anti-Gli2, anti-MDR1, anti-Bcl2, and anti-PCNA antibodies (cat. no. 18989-1-AP, 22336-1-AP, 12789-1-AP, and 10205-2-AP) were obtained from Proteintech (Rosemont, IL, USA). EMD Millipore (Billerica, MA, USA) supplied the anti-GAPDH antibody (cat. No. MAB374). Cell-Light EdU Apollo 567 kits (cat. no. C10310-1) were purchased from Guangzhou RiboBio Co. (Guangzhou, China).

### Cell Culture and Establishment of DDP-Resistant Cell Lines

The human OC cell lines SKOV3 and ES-2 were obtained from the Chinese Academy of Sciences Cell Bank (Beijing, China), whereas A2780, A2780/DDP, and HEK293T cells were obtained from the American Type Culture Collection (Manassas, VA, USA). All cell lines were maintained at 37°C in a humidified atmosphere containing 5% CO_2_ in Dulbecco’s Modified Eagle’s Medium (cat. no. C11995500BT; Thermo Fisher Scientific) supplemented with 10% fetal bovine serum and 10 U/mL penicillin/streptomycin. Experiments were conducted using exponentially expanding cells. To produce DDP-resistant cells (SKOV3/DDP), escalating dosages of DDP, beginning at 50 ng/mL, were added to the culture media. Briefly, after the cells were treated with DDP (three-day treatment cycles), the culture medium was replaced with fresh medium devoid of drug for the following three days or one week until the cells recovered. Following the third cycle, the DDP dose was increased to a maximum of 1 µg/mL.

### Lentivirus Transfection

The specific method of lentivirus (LV) transfection is detailed in our previous study (25).

### Cell Transfection

Prof. Shiwen Luo and Prof. Yong Li of the First Affiliated Hospital of Nanchang University (Nanchang, China) generously donated the pCMV6-Entry-*Gli2*-Myc vector carrying the human *Gli2* sequence (GenBank accession no. NM_005270) and corresponding empty vector (26). The SKOV3/DDP cells were seeded into a 6-well plate and cultured until a confluence of 70–80% was reached. According to the manufacturer’s protocol, Lipofectamine 2000 was used to transfect SKOV3/DDP cells with 2 µg of either pCMV6-Entry-*Gli2*-Myc or empty plasmids. After 3–6 h, the culture medium was replaced, and the cells were cultured for additional 24–48 h.

### Cell Viability and Chemosensitivity Assay

SKOV3/DDP, A2780/DDP, and ES-2 cells were seeded into 96-well plates (2,000 cells/100 µL medium/well). After 24 h culture, the medium was replaced with fresh medium containing varying concentrations of DDP (0–10 µg/M for SKOV3/DDP, 0–64 µg/M for A2780/DDP) and the procedure was repeated in triplicate. Untreated cells served as negative controls. After 48 h, viability was assessed using the CCK-8 assay according to the manufacturer’s instructions, and optical density was measured using an automated microplate reader. The 50% maximum inhibitory concentration (IC_50_), which is the concentration at which cell growth decreased by 50% compared with control cell growth, was calculated using the GraphPad Prism 7.0 software (GraphPad, Inc., San Diego, CA, USA).

### Cell Proliferation Assays

Logarithmic growing cells were collected and seeded into 96-well plates (8,000 cells/well). After 48 h, the medium was replaced with fresh medium containing 50 μM 5-ethynyl-2′-deoxyuridine (EdU), and the cells were incubated at 37°C for 1 h. The cell-Light EdU experiment was conducted according to the manufacturer’s instructions. Three biological replicates of each treatment were performed, and three fields of cells were counted in each well. Images were obtained using an inverted fluorescent microscope (IX71; Olympus, Tokyo, Japan), and data were analyzed using the ImageJ software (https://imagej.nih.gov/ij/index.html; National Institutes of Health, Bethesda, MD, USA).

### Western Blot (WB) Analysis

RIPA buffer and the bicinchoninic acid kit were used for total protein extraction and quantification, respectively. The samples denatured at 100°C for 10 min, and 20–100 µg of protein/sample was electrophoretically separated on a 8–12% polyacrylamide gel. The proteins were then transferred onto a nitrocellulose membrane (cat. no. IPVH00010; EMD Millipore). The membrane was blocked with 5% skimmed milk for 1 h at 4°C, and then incubated for 12–16 h at 4°C with the following primary antibodies: rabbit polyclonal anti-Gli2 (1:500 dilution), rabbit polyclonal anti-MDR1 (1:1,000 dilution), rabbit polyclonal anti-γH2AX (1:2,500 dilution), rabbit polyclonal anti-PCNA (1:5,000 dilution), rabbit polyclonal anti-Bcl2 (1:2,000 dilution), and mouse monoclonal anti-GAPDH (1:2,500 dilution). Then, the membrane was incubated with horseradish peroxidase-conjugated anti-rabbit and anti-mouse secondary antibodies at 37°C for 30 min. The protein bands were visualized by exposing the membrane to X-ray film, and the band density was analyzed using the ImageJ software. Each experiment was performed at least three times.

### Dual-Luciferase Reporter Assay

The promoter region of *MDR1* was identified based on data obtained from the National Center for Biotechnology Information (https://www.ncbi.nlm.nih.gov/). Moreover, the JASPAR tool (http://motifmap.ics.uci.edu/) was used to predict the *Gli2*-binding site (GBS) in the *MDR1* sequence. A human genomic DNA template was used to amplify the human *MDR1* promoter, which was then cloned into the pGL4.20 empty vector (Promega, Madison, WI, USA). To further evaluate the functional GBS, we used a mutagenesis kit (SMK-101, Toyobo, Osaka, Japan) to introduce a point mutation in the predicted GBS. The luciferase reporter gene plasmid was successfully constructed and co-transfected into HEK293T cells as follows: *Gli2* expression plasmid (0.5 μg), pGL4.20-*MDR1*-luci luciferase reporter gene plasmid (0.5 μg), and pRL-TK plasmid used as internal reference (0.025 μg). Studies have shown that the activation of the Bcl2 promoter in response to Hh/Gli signal transduction is predominantly mediated by Gli2 (27). Therefore, we used *Bcl2* as a positive control. After 48 h, the cells were collected and analyzed according to the manufacturer’s instructions using the dual-luciferase reporter gene detection system (Promega). The luciferase activity was standardized to the respective Renilla luciferase activity (internal control). Each transfection experiment was repeated at least three times, and each sample was examined three times in duplicate.

### *In Vivo* Ovarian Cancer Xenotransplant

All animal-related experiments were performed in accordance with the Guidelines for the Care and Use of Experimental Animals and authorized by the Nanchang University Institutional Animal Care and Use Committee and regional authorities. Nude mice (6–8 weeks old, 16–18 g; n = 10) were purchased from SLAC Laboratory Animal Co. (Hunan, China). To investigate the effect of *Gli2* signaling on DDP resistance in OC *in vivo*, 1 × 10^7^ SKOV3 sh- control or sh-*Gli2* cells were injected into the flanks of the mice. Tumor growth began four days later. After the tumor was observed, DOX was added to the drinking water of mice at a final concentration of 2 mg/mL. The xenografted tumor size and mouse body weight were measured every two days. The tumor volume was calculated according to the following formula: 0.5 × L × W^2^, where L is the tumor size at the longest point and W is the tumor size at the widest point. To evaluate drug resistance *in vivo*, xenotransplanted mice were randomly allocated to one of two groups (n = 5 per group) to receive intraperitoneal injections of DDP (4 mg/kg) or PBS (control group) twice per week for 12 days. On day 32, the animals were euthanized with an anesthetic overdose and the tumors were resected, weighed, and stored until further analysis.

### Immunohistochemical (IHC) Analysis

Tumor tissues collected from mice were fixed in 10% neutral-buffered formalin solution, dried, and embedded in paraffin blocks, which were subsequently cut into 3-μm-thick sections for IHC staining (28).

### Statistical Analysis

All statistical analyses were performed using the GraphPad Prism 7.0 software. The unpaired Student’s *t*-test was performed for comparisons between two experimental groups, and one-way analysis of variance was used for comparisons among three or more groups. Unless otherwise specified, all data from biological triplicates were averaged, and values are presented as the means ± standard deviation. A *P* value of < 0.05 was considered statistically significant. The ImageJ software was used to quantify the experimental data.

## Results

### Gli2 Expression Is Upregulated in DDP-Resistant OC Cells

**Figure 1:**
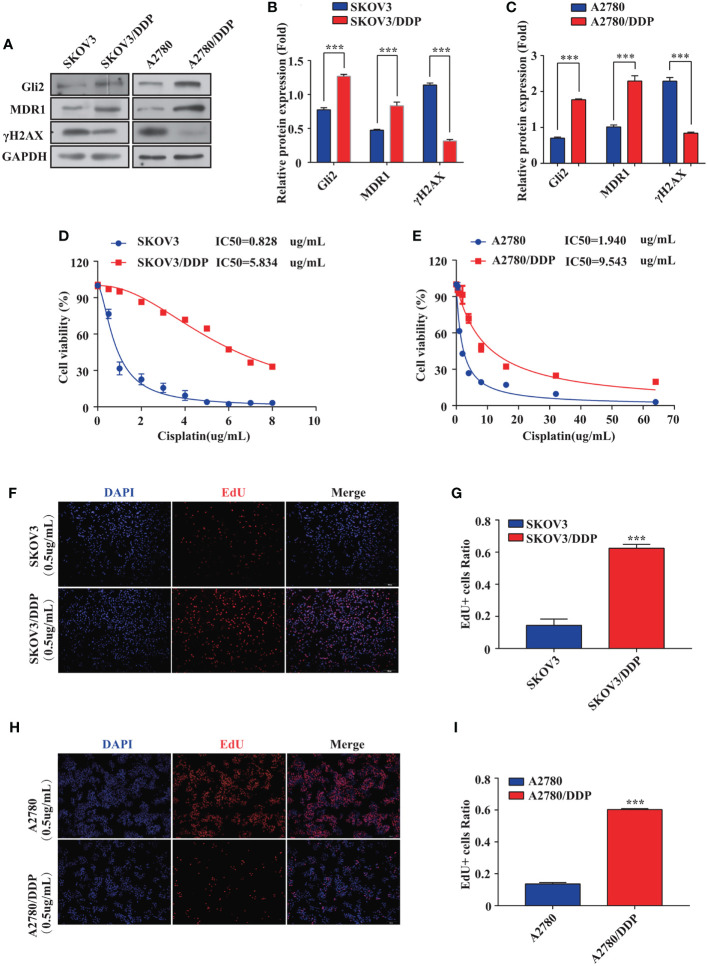
Gli2 is involved in ovarian cancer chemoresistance. **(A–C)** Western blot analysis of the expression of Gli2, MDR1 and γH2AX in SKOV3, SKOV3/DDP, A2780, and A2780/DDP cells. **(D, E)** Survival rates of SKOV3, SKOV3/DDP, A2780, and A2780/DDP cells after treatment with cisplatin (DDP) for 48 h detected using CCK-8 assays. **(F, H)** Proliferative capacity of SKOV3, SKOV3/DDP, A2780, and A2780/DDP cells after treatment with DDP for 48 h was identified using 5-ethynyl-2′-deoxyuridine (EdU) assays. **(G, I)** Bar chart of EdU-positive cells evaluated using the ImageJ software. ***P < 0.001.

First, stepwise selection of SKOV3 cells cultured in growth media with increasing DDP concentrations was performed to establish DDP-resistant OC cells (SKOV3/DDP). The IC_50_ values of wild-type SKOV3 and SKOV3/DDP cells grown for approximately six months in media containing 1 µg/mL DDP were compared. Notably, a 7-fold increase in the IC_50_ of DDP was observed for SKOV3/DDP cells compared with that for wild-type cells (**Figure 1D**), confirming the successful establishment of DDP-resistant OC cells. A2780 and A2780/DDP cells are well-known paired DDP-sensitive and DDP-resistant cell lines (29). Compared with A2780 cells, A2780/DDP cells had a 5-fold increase in DDP resistance (**Figure 1E**). The expression of Gli2 was upregulated and the expression of MDR1 was increased in SKOV3/DDP and A2780/DDP cells, whereas the level of DNA damage-associated γH2AX was reduced (**Figures 1A–C**). These results suggest that dysregulated transcription factor Gli2 is involved in OC DDP resistance. Next, the cells were treated with 0.5 µg/mL DDP for 48 h and EdU proliferation experiments were performed. Compared with the parental SKOV3 and A2780 cell lines, the proliferative ability of SKOV3/DDP and A2780/DDP cells was significantly enhanced (**Figures 1F–I**).

### *Gli2* Knockdown Reverses DDP Resistance and Inhibits Proliferation of DDP-Resistant OC Cells

**Figure 2:**
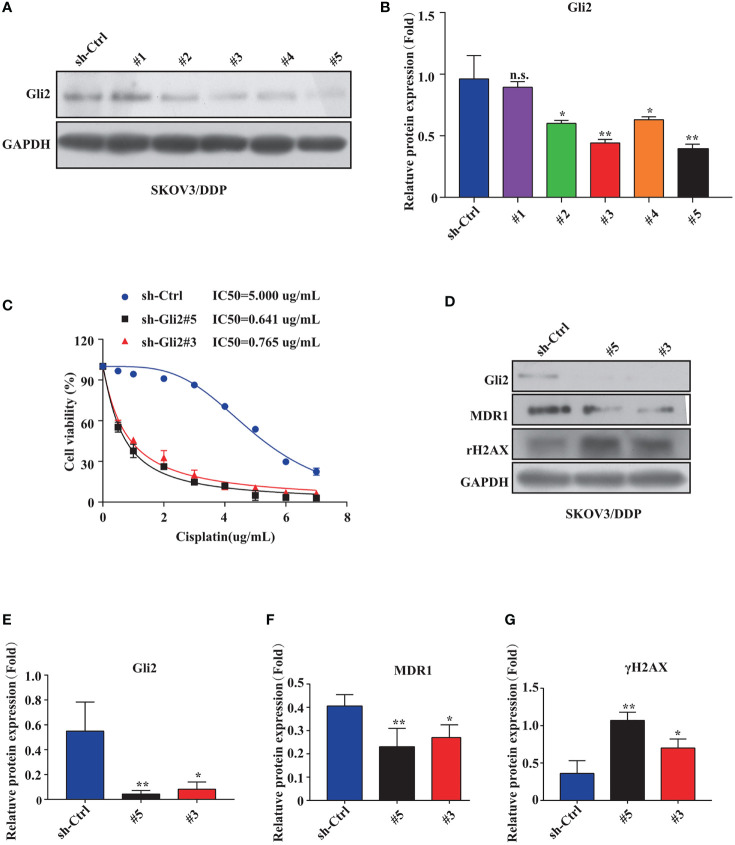
*Gli2* knockdown reversed cisplatin (DDP) resistance and inhibited proliferation of DDP-resistant ovarian cancer cells. **(A, B)** Western blot analysis of the protein expression levels of Gli2 in different groups after *Gli2* knockdown in SKOV3/DDP cells. **(C)** Survival rate and IC_50_ of SKOV3/DDP cells transfected with sh-control or sh-Gli2 #3 and #5 after 48 h DDP treatment determined using CCK-8 assays. **(D–G)** Western blot analysis of the expression levels of MDR1 and γH2AX in SKOV3/DDP cells transfected with empty plasmid or *Gli2* specific short hairpin RNA following DDP treatment (0.5 µg/mL). n.s, not significant, * *P* < 0.05, **P < 0.01.

The impact of inhibiting *Gli2* on OC cell DDP resistance was evaluated. SKOV3/DDP cells were transfected with *Gli2* short hairpin RNAs (sh-*Gli2* #1–5), which significantly downregulated *Gli2* expression in SKOV3/DDP cells, particularly sh-*Gli2* #3 and #5, as compared with sh-control transfected cells (**Figures 2A, B**). Therefore, cells transfected with sh-*Gli2* #3 and #5 were selected for further analysis.

**Figure 3:**
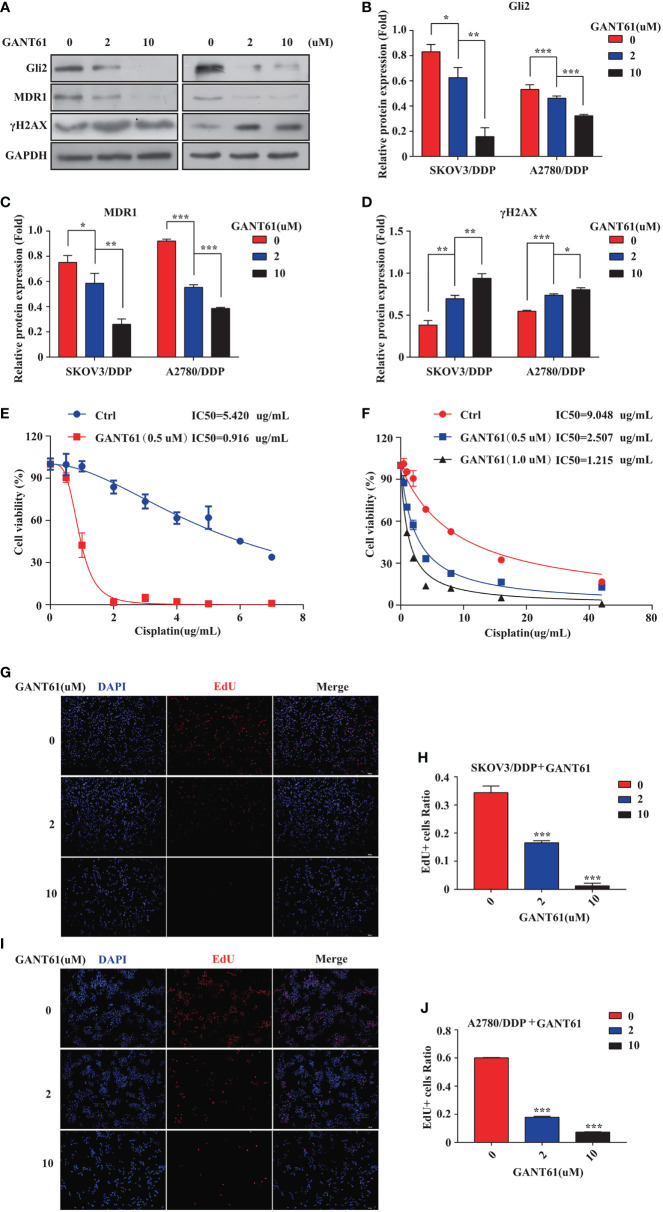
The Gli2 inhibitor promoted cell drug sensitivity and reduced the proliferative ability of cisplatin (DDP)-resistant ovarian cancer cells. **(A–D)** Western blot analysis of the protein expression levels of Gli2, MDR1, and γH2AX in SKOV3/DDP and A2780/DDP cells cultured with increasing concentrations of the Gli2 inhibitor Gli-antagonist 61 (GANT61) for 48 h. **(E, F)** Survival rate and IC_50_ of SKOV3/DDP and A2780/DDP cells after 48 h of treatment with GANT61 and DDP determined using CCK-8 assays. **(G–J)** Proliferation capacity was detected by EdU assay after inhibition of SKOV3/DDP and A2780/DDP cells. *P < 0.05, **P < 0.01, ***P < 0.001.

The mechanism of action of DDP is well-known. DDP induces DNA damage by combining with DNA molecules to form platinum-DNA adducts, leading to cytotoxicity and initiating apoptosis. DNA damage-induced apoptosis is an important anti-tumor mechanism of various drugs (30). γH2AX is a biomarker of DNA damage. When DNA is damaged, the expression of γH2AX is increased, whereas following damage repair, its expression is downregulated. Thus, the effect of sh-*Gli2* on the cytotoxic effects of DDP in SKOV3/DDP cells was assessed by analyzing γH2AX expression. Furthermore, to detect the cytotoxic effects of DDP, a CCK-8 viability assay was performed. Overall, *Gli2* knockdown significantly improved the efficacy of DDP treatment. The IC_50_ value for DDP in SKOV3/DDP cells (**Figure 2C**) decreased from 5.000 µg/mL for SKOV3/DDP cells to 0.641 and 0.765 µg/mL for sh-*Gli2* #5 and #3 transfected cells, respectively, suggesting that *Gli2* knockdown enhanced the sensitivity of SKOV3/DDP cells to DDP. Moreover, the WB results showed that *Gli2* knockdown downregulated the expression of MDR1 in SKOV3/DDP cells compared with that in control cells. Because of the increased sensitivity of cells to DDP after downregulation of *Gli*2 expression, which resulted in increased DNA damage, the expression of γH2AX was significantly upregulated (**Figures 2D–G**). Consistently, similar results were obtained when *Gli2* expression was inhibited with the Hh signaling pathway inhibitor GANT61, which specifically inhibits *Gli*, in SKOV3/DDP and A2780/DDP cells (**Figures 3A–F**). Moreover, EdU analysis showed that downregulation of *Gli2* markedly inhibited the proliferation of SKOV3/DDP and A2780/DDP cells (**Figures 3G–J**). Collectively, these data indicate that inhibition of *Gli2* expression reduces DDP resistance in DDP-resistant OC cells.

### MDR1 Is Essential for DDP Resistance in OC

**Figure 4:**
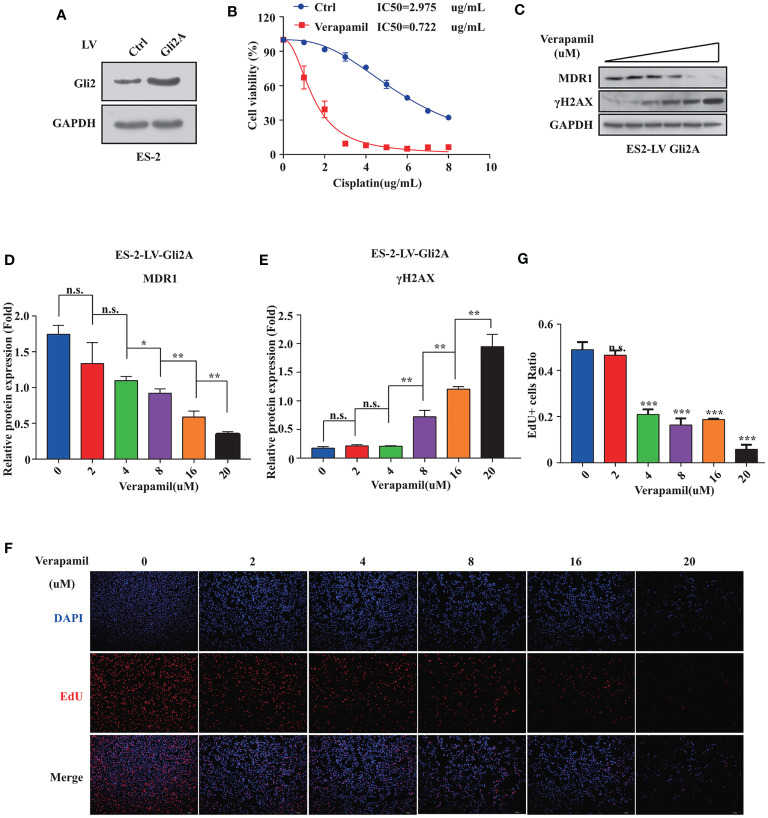
Reversal of cisplatin (DDP) resistance in ovarian cancer cells following treatment with MDR1 inhibitor verapamil. **(A)** Successful construction of stable cell line overexpressing *Gli2* using lentiviral infection. **(B)** Survival rate and IC_50_ of SKOV3/DDP cells after treatment with verapamil and DDP for 48 h determined using CCK-8 assays. **(C–E)** Western blot analysis of the protein expression levels of MDR1 and γH2AX in cells treated with gradient concentrations of verapamil for 48h. **(F–G)** Downregulation of MDR1 expression significantly inhibited cell proliferation, as detected using EdU assays. n.s., not significant, *P < 0.05, **P < 0.01, ***P < 0.001.

To further explore if *MDR1* inhibition could reverse DDP resistance in OC cells, an ES-2 cell line stably overexpressing *Gli2* was established (**Figure 4A**). Verapamil, a calcium channel-blocker and anti-arrhythmic drug, is a competitive inhibitor of MDR1 that does not directly compete for active site binding with MDR1 substrates but influences protein activity *via* allosteric inhibition (31–33). ES-2-LV-*Gli2* cells treated with 5 μM verapamil showed a marked reversal of DDP resistance (**Figure 4B**), along with a 4-fold reduction in the IC_50_ value for DDP. Notably, after gradient addition of verapamil (0, 2, 4, 8, 16, and 20 μM) to the culture medium for combination treatment with DDP (0.5 µg/mL), the sensitivity of ES-2-LV-*Gli2* cells to DDP increased with the increasing verapamil concentration compared with that in control cells. The WB results further showed that *MDR1* expression was significantly downregulated and that DDP-induced DNA damage had increased (**Figures 4C–E**). Moreover, downregulation of *MDR1* significantly inhibited ES-2-LV-*Gli2* cell proliferation (**Figures 4F, G**). Thus, our results provided evidence that *MDR1* expression is essential for DDP resistance.

### *MDR1* Is a Direct Downstream Target of *Gli2*

**Figure 5:**
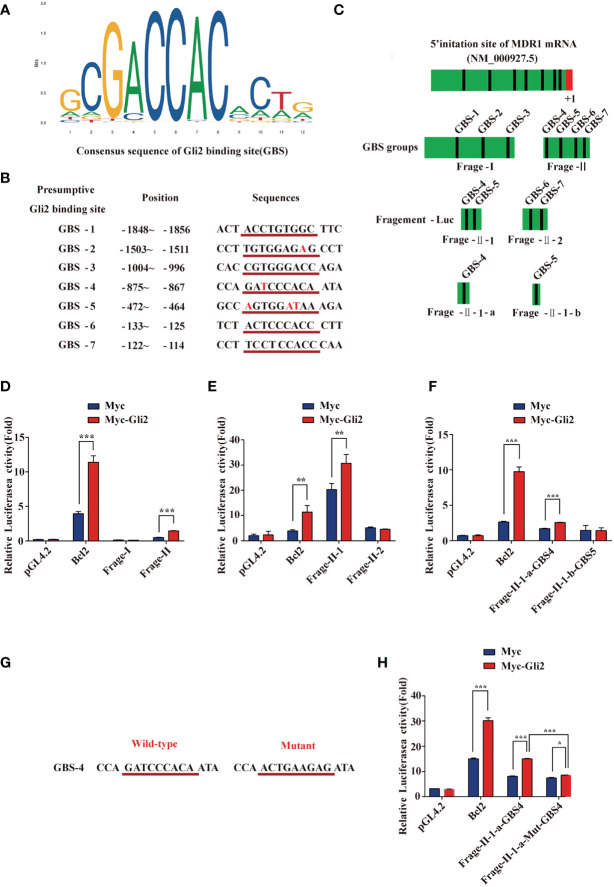
*MDR1* is the direct downstream target of *Gli2*. **(A)** Consensus sequence of *Gli2*-binding sites (GBS). The size of each base in the figure indicates its conservation. The larger the base, the stronger the conservation and more important its function. **(B)** Within the range of -2,002 to 0 in the promoter region of *MDR1* and adjacent regions (the first base at the 5′ end of the MDR1 mRNA NM_000927.5 was recorded as +1), seven candidate GBS were predicted using software analysis. They are numbered separately, and the position and sequence are indicated. The consensus sequence with GBS is underlined in red with the corresponding sequence, and the base list differing from the GBS consensus sequence is in red font. **(C)** Schematic illustration of the distribution of candidate GBS within the *MDR1* promoter and luciferase reporter constructs Frag-I, -II, –II-1, –II-2, –II-1-a, and –II-1-b containing the indicated GBS. **(D–F)** Gli2 activating effects on Frag-I, -II, –II-1, –II-2, –II-1-a, and –II-1-b reporter constructs detected using a dual-luciferase assay in *Gli2*-transfected and control HEK293 cells. **(G)** Wild-type and mutant sequences of the GBS-4 locus in Frag–II-1-a. **(H)** Compared with the wild-type, the activation effect of Gli2 on the mutant Frag–II-1-a was significantly reduced after GBS-4 point mutation. “Mut” indicates a mutation, and the letters after it denote different GBS sites. Data are represented as the mean ± SD of three independent experiments. n = 3, *P < 0.05, **P < 0.01, ***P < 0.001.

Our previous studies indicated that *MDR1* is the downstream target of *Gli2 (* 25). Moreover, the results described above also demonstrate that *Gli2* positively modulates the expression of *MDR1* in drug-resistant OC cells. To further explore whether *Gli2* directly regulates *MDR1* expression to promote DDP resistance in OC, the JASPAR tool (http://motifmap.ics.uci.edu/) was used to predict the GBS in the *MDR1* sequence (**Figure 5A**). Seven potential *Gli2*-binding sites were identified within the −2,002–0 genomic region (the 5′ initiation site of *MDR1* [NM_000927.5] was numbered +1) (**Figure 5B**). Different truncated *MDR1* promoter reporter sequences were constructed for all seven putative GBS genomic sequences and cloned into the pGL4.20 plasmid to produce six luciferase reporter gene constructs, namely fragment 1 (Frag-I) and fragment 2 (Frag-II), with the latter being continuously segmented (Frag–II-1, Frag–II-2, Frag–II-1-a, and Frag–II-1-b) (**Figure 5C**).

These constructs and *Gli2*, as well as pRL-TK (for normalization), were co-transfected into HEK293T cells with *Bcl2* or pCMV-Myc as a control. Cells were collected at 48 h after transfection, and a dual-luciferase reporter assay was performed. The relative luciferase activity was obtained by normalizing the firefly luciferase activity against the internal Renilla luciferase activity. The luciferase assay results showed that the relative luciferase activity of the *MDR1* promoter increased only in specific DNA sequences (GBS-4: −875 to −867) compared with that of a promoter-less control (**Figures 5D–F**). We next used the point mutation method to mutate the GBS-4 sequence (**Figure 5G**), which significantly abolished the activation of the *MDR1*-Luci reporter by *Gli2* (**Figure 5H** **)**, revealing that *MDR1* is a direct downstream target of *Gli2*.

### *Gli2* Knockdown Overcomes DDP Resistance in OC *In Vivo*

**Figure 6:**
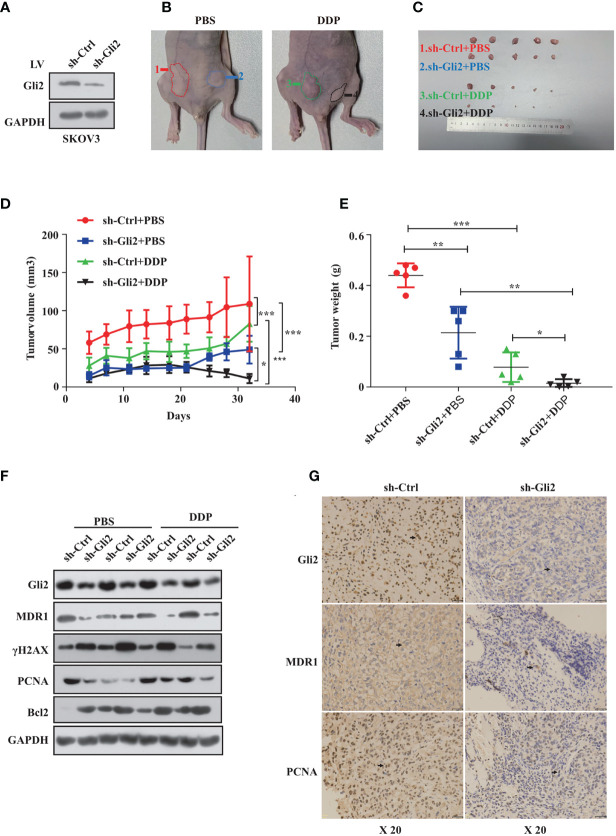
*Gli2* knockdown trumps cisplatin (DDP) resistance in ovarian cancer *in vivo*. SKOV3 cells infected with sh-Control or sh-*Gli2* were subcutaneously injected into nude mice. **(A)** Western blot analysis of the Gli2 protein expression after stable knockdown of *Gli2* in SKOV3 cells. **(B, C)** SKOV3 cells infected with sh-Control or sh-*Gli2* were subcutaneously injected into the skin of nude mice. The mice were injected intraperitoneally with 4 mg/kg DDP or phosphate-buffered saline (PBS) (control group) twice per week and photographed on day 32. Tumor volume **(D)** and tumor weight **(E)** data are shown as the mean ± SD (n = 5). **(F)** Following *Gli2* knockdown, the expression levels of MDR1 and PCNA decreased, while those of γH2AX and Bcl2 increased *in vivo*. **(G)** Expression of Gli2, MDR1, and PCNA in SKOV3 sh-control and SKOV3 sh-Gli2 tumors determined using immunohistochemical analysis. *P < 0.05, **P < 0.01, ***P < 0.001.

Next, we established a SKOV3 cell line that stably reduced *Gli2* expression. Analysis of total protein lysates by WB confirmed that the *Gli2* levels were reduced (**Figure 6A**). These cells were then transplanted into mice to test the impact of *Gli2* expression on the tumorigenicity and drug resistance of SKOV3 cells *in vivo*. SKOV3-sh-control and SKOV3-sh-*Gli2* cells were subcutaneously injected into nude mice, which were then randomly assigned for treatment with intraperitoneal injections of PBS (control group) or DDP (treated group). Tumors formed by SKOV3 cells lacking *Gli2* were smaller and lighter than those formed by cells expressing normal levels of *Gli2* (**Figures 6B, C**). Moreover, DDP treatment alone showed a limited ability to inhibit tumor growth in cells expressing normal levels of *Gli2* as compared with the control group because of DDP resistance. In contrast, combining *Gli2* knockdown with DDP treatment caused a marked decrease in the tumor volume and mass. Tumor xenografts developed slowly (**Figure 6D**) in the sh-*Gli2* group compared to the sh-control group, resulting in smaller tumor xenografts (**Figure 6E**). Taken together, these results suggest that knocking down *Gli2* can inhibit DDP resistance and enhance the therapeutic effect of DDP on OC *in vivo*. In accordance with the *in vitro* data, further detailed analysis of the tumor tissues revealed that the MDR1 and proliferating cell nuclear antigen (PCNA) protein levels were decreased, whereas the γH2AX and apoptosis protein Bcl2 levels were upregulated in the mouse tumors upon *Gli2* knockdown (**Figures 6F, G**). These results strongly indicate that *Gli2* is an important regulator of OC cell drug resistance *in vivo*.

## Discussion

**Figure 7:**
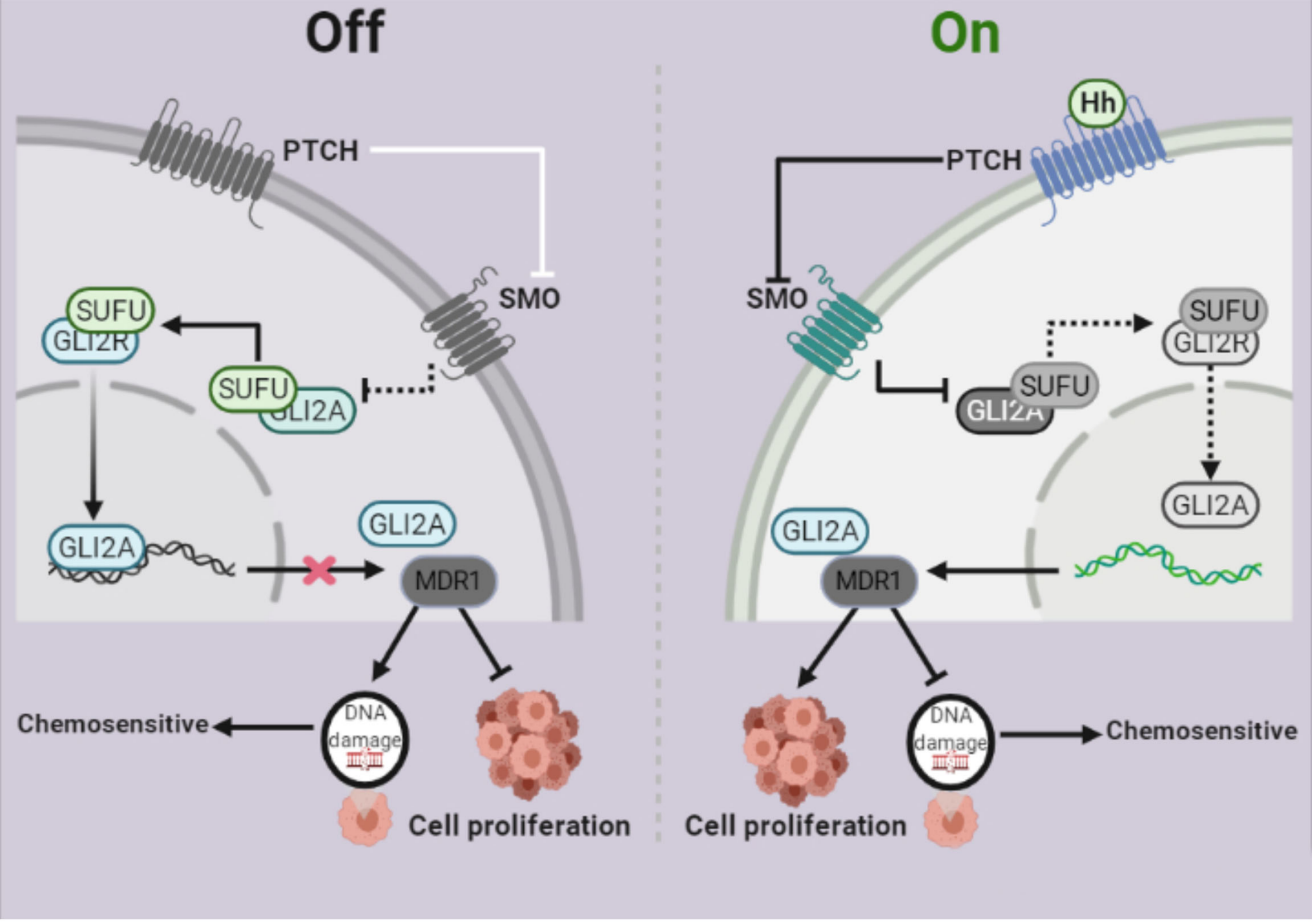
Schematic depiction of the role of Gli2 in cisplatin (DDP) resistance in ovarian cancer. Under normal circumstances, the Hh signaling pathway is inhibited and therefore, Gli2 cannot bind to the downstream target genes. This maintains DDP sensitivity in ovarian cancer cells and cell proliferation is inhibited. When the Hh signaling pathway is abnormally activated, Gli2 is overexpressed, enters the nucleus in the activated form of Gli2A and directly binds to the downstream target gene *MDR1.* This promotes DDP resistance in ovarian cancer cells, thereby significantly enhancing cell proliferation.

The Hh signaling system has been evolutionarily preserved (34). Several studies have shown that Hh signaling is intimately linked to the formation and progression of a variety of malignant tumors (35, 36). Despite significant advancements in the treatment of ovarian cancer, chemoresistance remains the leading cause of therapy failure and death from this disease. Some studies showed that *Gli1* is linked to tumor resistance (37, 38), whereas others suggested that *Gli2*, rather than *Gli1*, may be more predictive of resistance (39). For example, Steg AD et al. found that by targeting the Hh signaling pathway, transcription factor Gli2 increased sensitivity to DDP in ovarian cancer (40). However, our findings showed that *Gli2* expression is involved in DDP resistance in OC. MDR1 is a drug efflux pump that plays an important role in chemoresistance. MDR1 effluxes different chemotherapeutic drugs from tumor cells (41, 42,) and its expression is adversely linked to the prognosis of various malignancies, including OC (43). This study demonstrated that MDR1 plays a vital role in OC cell chemoresistance and that it is regulated by *Gli2* (**Figure 7**).

First, DDP resistant SKOV3/DDP cells were established by stimulation with gradient concentrations of DDP. Further analysis showed that compared with the parental strains (SKOV3 and A2780), the IC_50_ value of DDP-resistant SKOV3/DDP and A2780/DDP cells increased by 7- and 5-fold, respectively, and that *Gli2* expression was increased. The *in vitro* results also showed that the sensitivity of SKOV3/DDP cells to DPP was significantly higher after transfection with *Gli2*-interfering plasmids or treatment with the Hh inhibitor GANT61, suggesting that *Gli2* expression promotes DDP resistance in OC cells. *MDR1* expression was also reduced, supporting that *MDR1* expression is regulated by *Gli2* in OC resistance. This is consistent with the results of Zhao et al (44). Furthermore, the findings of the current study support previously reported results (25). Studies have shown that certain chemotherapeutic drugs are more effective in tumor suppression, even in OC, when combined with verapamil (45, 46). We obtained comparable results by adding gradient concentrations of the MDR1 inhibitor verapamil to the stable *Gli2*-overexpressing cell line ES-2-LV-*Gli2*, suggesting that verapamil pretreatment effectively reversed drug resistance in a dose-dependent manner (45).

*In vitro* proliferation experiments by EdU showed that the proliferation ability of drug-resistant cells was significantly higher than that of the parental strains. Targeting *Gli2* reduced the proliferative ability of the cells. Furthermore, the dual-luciferase assay confirmed that *Gli2* regulates OC chemotherapy resistance by regulating *MDR1* transcription. Moreover, we demonstrated that *Gli2* knockdown can reverse the DDP resistance of OC cells in nude mice transplanted with tumor cells. Therefore, *Gli2* may promote OC chemotherapy resistance by regulating *MDR1* expression *in vivo and in vitro*.

Taken together, these results demonstrate that *Gli2* promotes OC chemotherapy resistance by regulating the expression of *MDR1* both *in vitro* and *in vivo*. Hence, *Gli2* and *MDR1* are potential novel therapeutic targets to increase the clinical efficacy of DDP in treating patients with OC. Furthermore, *Gli2* and *MDR1* are potential indicators for assessing DDP resistance during clinical OC treatment.

## Data Availability Statement

The original contributions presented in the study are included in the article/supplementary material. Further inquiries can be directed to the corresponding author.

## Ethics Statement

The animal study was reviewed and approved by the Nanchang University Institutional Animal Care.

## Author Contributions

QW, XW, LH, LZ, and HZ performed the experiments. QW analyzed the data and prepared the figures. QW and XW wrote the manuscript. QC and QW contributed to the study design, data interpretation, and final editing of the manuscript. All authors read and approved the final manuscript.

## Funding

This work was supported in part by grants from the National Natural Science Foundation of China (81960470 to QC) and Key R&D Project of Jiangxi Science and Technology Department (grant no. 20202ACB206008).

## Conflict of Interest

The authors declare that the research was conducted in the absence of any commercial or financial relationships that could be construed as a potential conflict of interest.

## Publisher’s Note

All claims expressed in this article are solely those of the authors and do not necessarily represent those of their affiliated organizations, or those of the publisher, the editors and the reviewers. Any product that may be evaluated in this article, or claim that may be made by its manufacturer, is not guaranteed or endorsed by the publisher.
